# Supplementary material for: Utilizing digital pathology to quantify stromal caveolin-1 expression in malignant and benign ovarian tumors: Associations with clinicopathological parameters and clinical outcomes
Source: PLoS One. 2021 Nov 23;16(11):e0256615. doi: 10.1371/journal.pone.0256615 (PMC8610269; doi:10.1371/journal.pone.0256615)
Supplement: S1 File — (DOCX) [file pone.0256615.s001.docx]

Supplementary IF staining:

The first step of the protocol consists of primary antibody incubation using a vimentin mouse monoclonal antibody (Abcam Biotechnology lot: ab8069, Cambridge, UK), to visualize the fibroblasts in the stroma, at a 1:80000 dilution for 30 minutes. This is followed by the labeled polymer incubation step using a mouse envision reagent (Dako ref: K4007) for 30 minutes and a substrate, TSA-Cy3 (PerkinElmer ref: NEL744B001KT), incubated at a 1:50 dilution for 5 minutes. After the first round of IF staining the slides were washed in a 0.1% azide solution and the staining process was repeated this time using a CAV-1 rabbit polyclonal antibody (Santa Cruz lot: G0314) at a 1:5000 dilution and a guinea pig pancytokeratin tumor masking antibody (Acris lot: 411101, Rockville, MA) at a 1:100 dilution for 30 minutes. This was followed by the labeled polymer incubation step using a rabbit envision reagent (Dako ref: K4011) and a secondary antibody, Alexa488 guinea pig (Abcam, ab150185), to visualize the tumor masking antibody, at a 1:200 dilution for 30 minutes and a substrate TSA-Cy5 (Perkin Elmer ref: NEL745B001KT) incubated at a 1:50 dilution for 5 minutes. The slides were cover-slipped with ProLong Gold antifade reagent with 4',6-diamidino-2-phenylindole (DAPI) mounting media to visualize the nuclei.
