# Supplementary material for: Utilizing digital pathology to quantify stromal caveolin-1 expression in malignant and benign ovarian tumors: Associations with clinicopathological parameters and clinical outcomes
Source: PLoS One. 2021 Nov 23;16(11):e0256615. doi: 10.1371/journal.pone.0256615 (PMC8610269; doi:10.1371/journal.pone.0256615)
Supplement: S1 Fig — (DOCX) [file pone.0256615.s003.docx]

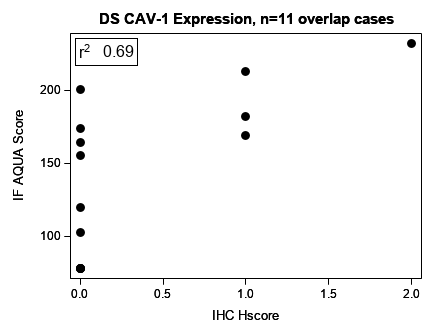

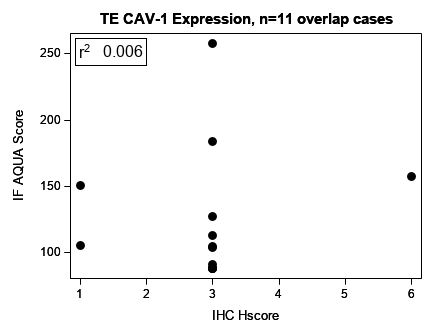

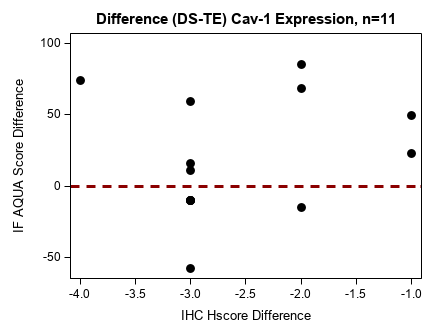


**Figure S1. Comparison of Cav-1 expression quantified by immunofluorescence and immunohistochemistry.**
