# Supplementary material for: Utilizing digital pathology to quantify stromal caveolin-1 expression in malignant and benign ovarian tumors: Associations with clinicopathological parameters and clinical outcomes
Source: PLoS One. 2021 Nov 23;16(11):e0256615. doi: 10.1371/journal.pone.0256615 (PMC8610269; doi:10.1371/journal.pone.0256615)
Supplement: S1 Table — (DOCX) [file pone.0256615.s004.docx]

Table S1: Association of Cav-1 expression with overall survival of serous ovarian cancer in Cohort 1 (N=73)

| **Independent Factors^a^** | **Univariate Estimates** | | **Cav1 Expression Adjusted Models^b^** | | | |
| --- | --- | --- | --- | --- | --- | --- |
|  |  |  | **Stroma Final Model** | | **Epithelial Final Model** | |
|  | **HR (95%CI)** | **p-value** | **HR (95%CI)** | **p-value** | **HR (95%CI)** | **p-value** |
| Age at diagnosis | 1.01 (0.99-1.03) | 0.32 | 1.02 (1.00-1.04) | 0.04 | 1.02 (1.00-1.04) | 0.04 |
| Response to Therapy | 7.27 (4.05-13.05) | <0.0001 | 8.28 (4.41-15.54) | <0.0001 | 8.61 (4.59-16.12) | <0.0001 |
| Nodal Status (pN) | 1.80 (0.87-3.72) | 0.11 | - | - | - | - |
|  |  |  |  |  |  |  |
| Stroma Cav-1 | 0.69 (0.45-1.07) | 0.09 | 0.83 (0.58-1.19) | 0.31 | NA | NA |
| Epithelium Cav-1 | 0.94 (0.80-1.10) | 0.41 | NA | NA | 0.99 (0.84-1.16) | 0.89 |

^a^ Response to therapy is modeled as Incomplete vs. Complete; Nodal status as Involved vs. Not Involved

^b^ Adjustment variables were forward selected for final model using P<0.10 as retained criterion.
