# Supplementary material for: Utilizing digital pathology to quantify stromal caveolin-1 expression in malignant and benign ovarian tumors: Associations with clinicopathological parameters and clinical outcomes
Source: PLoS One. 2021 Nov 23;16(11):e0256615. doi: 10.1371/journal.pone.0256615 (PMC8610269; doi:10.1371/journal.pone.0256615)
Supplement: S2 Table — (DOCX) [file pone.0256615.s005.docx]

Table S2: Association of Cav-1 expression with overall survival of serous ovarian cancer in Cohort 2 (N=105)

| **Independent Factors^a^** | **Univariate Estimates** | | **Cav1 Expression Adjusted Models^b^** | | | |
| --- | --- | --- | --- | --- | --- | --- |
|  |  |  | **Stroma Final Model** | | **Epithelial Final Model** | |
|  | **HR (95%CI)** | **p-value** | **HR (95%CI)** | **p-value** | **HR (95%CI)** | **p-value** |
| Age at diagnosis | 1.04 (1.01-1.06) | 0.002 | 1.06 (1.03-1.09) | <0.0001 | 1.06 (1.04-1.09) | <0.0001 |
| Response to Therapy | 3.00 (1.76-5.13) | <0.0001 | 3.85 (2.15-6.90) | <0.0001 | 3.79 (2.10-6.84) | <0.0001 |
| Stage | 10.95 (2.67-44.88) | 0.0009 | 13.62 (3.27-56.74) | 0.0003 | 13.75 (3.30-57.27) | 0.0003 |
| Tumor Size (pT) | 5.28 (2.11-13.21) | 0.0004 | - | - | - | - |
| Nodal Status (pN) | 2.40 (1.06-5.46) | 0.04 | - | - | - | - |
| Debulking Status | 1.94 (1.12-3.36) | 0.02 | - | - | - | - |
|  |  |  |  |  |  |  |
| Stroma Cav-1 | 1.01 (1.00-1.01) | 0.10 | 1.01 (1.00-1.01) | 0.37 | NA | NA |
| Epithelium Cav-1 | 1.00 (1.00-1.01) | 0.92 | NA | NA | 1.00 (1.00-1.01) | 0.81 |

^a^ Response to therapy is modeled as Incomplete vs. Complete; Stage as III/IV vs. I/II; Tumor Size (pT) as >50mm vs. <=50mm; Nodal status as Involved vs. Not Involved; Debulking as Suboptimal vs. Optimal.

^b^ Adjustment variables were forward selected for final model using P<0.10 as retained criterion.
