# Supplementary material for: Utilizing digital pathology to quantify stromal caveolin-1 expression in malignant and benign ovarian tumors: Associations with clinicopathological parameters and clinical outcomes
Source: PLoS One. 2021 Nov 23;16(11):e0256615. doi: 10.1371/journal.pone.0256615 (PMC8610269; doi:10.1371/journal.pone.0256615)
Supplement: S3 Table — (DOCX) [file pone.0256615.s006.docx]

Table S3: Association of tumor pathological features with overall survival of serous ovarian cancer (N=75)

| **Independent Factors^a^** | **Univariate Estimates** | | **Cav1 Expression Adjusted Models^b^** | | | |
| --- | --- | --- | --- | --- | --- | --- |
|  |  |  | **Stroma Final Model** | | **Epithelial Final Model** | |
|  | **HR (95%CI)** | **p-value** | **HR (95%CI)** | **p-value** | **HR (95%CI)** | **p-value** |
| Pathological features |  |  |  |  |  |  |
| TIL | 0.91 (0.52-1.58) | 0.72 | - | - | - | - |
| Cystic component | 1.02 (0.56-1.85) | 0.96 | - | - | - | - |
| LVI | **3.56 (1.51-8.38)** | **0.004** | **3.11 (1.30-7.45)** | **0.01** | **3.70 (1.47-9.28)** | **0.005** |
| Response to Therapy | **2.21 (1.20-4.06)** | **0.01** | **3.82 (1.90-7.71)** | **0.0002** | **4.07 (2.03-8.18)** | **<0.0001** |
| Stage | **9.86 (1.36-71.55)** | **0.02** | - | - | - | - |
| Age at diagnosis | **1.04 (1.01-1.07)** | **0.003** | **1.07 (1.03-1.10)** | **<0.0001** | **1.07 (1.03-1.10)** | **<0.0001** |
| Tumor Size (pT) | **5.3 (2.11-13.21)** | **0.0004** | **5.36 (1.28-22.52)** | **0.02** | **5.23 (1.25-21.84)** | **0.02** |
| Nodal Status (pN) | 2.19 (0.91-5.24) | 0.08 |  |  |  |  |
| Debulking Status | 1.51 (0.82-2.81) | 0.19 | **-** | **-** | **-** | **-** |
|  |  |  |  |  |  |  |
| Stroma Cav-1 | 1.01 (1.00-1.01) | 0.09 | 1.01 (1.00-1.01) | 0.18 | NA | NA |
| Epithelium Cav-1 | 1.00 (1.00-1.01) | 0.17 | NA | NA | 1.00 (1.00-1.01) | 0.12 |

^a^ Pathological features are modeled as Yes vs No; Response to therapy as Incomplete vs. Complete; Stage as III/IV vs. I/II; Tumor Size (pT) as >50mm vs. <=50mm; Nodal status as Not Involved vs. Involved; Debulking as Suboptimal vs. Optimal.

^b^ Adjustment variables were forward selected for final model using P<0.10 as retained criterion.
